# Demonstration of a robust magnonic spin wave interferometer

**DOI:** 10.1038/srep30268

**Published:** 2016-07-22

**Authors:** Naoki Kanazawa, Taichi Goto, Koji Sekiguchi, Alexander B. Granovsky, Caroline A. Ross, Hiroyuki Takagi, Yuichi Nakamura, Mitsuteru Inoue

**Affiliations:** 1 Department of Electrical and Electronic Information Engineering, Toyohashi University of Technology, 1-1 Hibari-Ga-Oka, Tempaku, Toyohashi, Aichi 441-8580, Japan; 2 JST, PRESTO, 4-1-8 Honcho, Kawaguchi, Saitama, 332-0012, Japan; 3 Department of Physics, Keio University, Yokohama 223-8522, Japan; 4 Faculty of Physics, Moscow State University, Leninskie Gory, Moscow 119992, Russia; 5 Department of Materials Science and Engineering, Massachusetts Institute of Technology, 77 Massachusetts Avenue, Cambridge, Massachusetts 02139, USA

## Abstract

Magnonics is an emerging field dealing with ultralow power consumption logic circuits, in which the flow of spin waves, rather than electric charges, transmits and processes information. Waves, including spin waves, excel at encoding information via their phase using interference. This enables a number of inputs to be processed in one device, which offers the promise of multi-input multi-output logic gates. To realize such an integrated device, it is essential to demonstrate spin wave interferometers using spatially isotropic spin waves with high operational stability. However, spin wave reflection at the waveguide edge has previously limited the stability of interfering waves, precluding the use of isotropic spin waves, i.e., forward volume waves. Here, a spin wave absorber is demonstrated comprising a yttrium iron garnet waveguide partially covered by gold. This device is shown experimentally to be a robust spin wave interferometer using the forward volume mode, with a large ON/OFF isolation value of 13.7 dB even in magnetic fields over 30 Oe.

A spin wave (SW) is a radio frequency (rf) collective excitation of the magnetic moments in a magnetic material, a magnetic counterpart of elastic waves. The transmission properties of SWs can be extensively modulated depending on the strength of the bias magnetic field, the waveguide material and the geometry^1^,^2^. This enables engineering of arbitrary band structures for carrier signals in the several-GHz range. These unique properties have been widely used to design passive radio frequency elements, including tunable filters^3^ and delay lines^4^. However, the most important feature of SWs is their propagation without charge transport. Thus SWs have attracted attention because they offer a new paradigm for information processing in which Joule loss and its accompanying heat generation is expected to be extremely small. Therefore, SWs could be used to represent information in beyond-CMOS devices^5^. To develop logic circuits based on SWs, active control of SW flow is required. Recently, a variety of systems showing spin dynamic effects^6^,^7^,^8^,^9^,^10^,^11^,^12^ and artificially designed structures^13^,^14^,^15^,^16^,^17^,^18^ manipulating the local magnetic moments have been investigated. These novel systems have motivated the creation of *magnonics* as a research field that addresses transport, storage and processing information using SWs^19^,^20^. A significant feature of SWs compared to other information carriers is the usefulness of their phase information, which can be easily manipulated in logic operations. Therefore a SW interferometer is an essential component for realization of logic devices^21^,^22^,^23^.

Yttrium iron garnet (YIG) is suitable for SW waveguides because of its low magnetic damping and low out-of-plane saturation magnetic field^24^,^25^,^26^,^27^,^28^. Magnons can therefore conserve their phase information over long distances in a YIG waveguide. Yu *et al.* recently experimentally observed SW propagation over 600 μm using a 20 nm-thick YIG waveguide with in-plane magnetization^29^. Not only the attenuation length, but also the magnetization configuration is crucial in determining the propagation mode of the SW. Unlike backward volume (BV) and Damon-Eshbach (DE) SWs with in-plane magnetization, forward volume (FV) SWs with out-of-plane magnetization can propagate through waveguides in arbitrary directions^30^. This isotropy of the FV SWs makes them suitable for integrated devices. However, the isotropic wave also generates extra standing waves which disrupt the control of the SW flow. In this work a SW absorber comprising a metallized YIG waveguide^31^ was designed and fabricated for the FV mode based on numerical results, and a SW interferometer was demonstrated with high operational stability.

## Results and Discussion

### Concept and experimental setup

**Figure 1:**
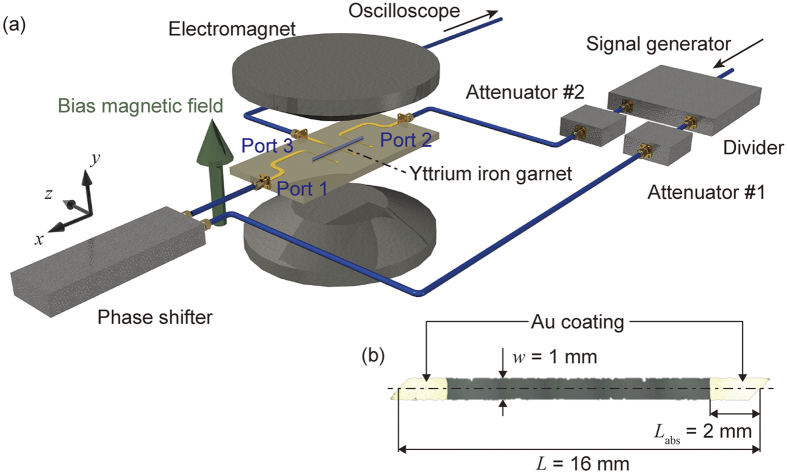
Setup for FV SW interferometer. (**a**) Experimental system. Port 1 and port 2 are injection ports. Port 3 is the detection port. (**b**) Top view of the SW waveguide, which was cut into a parallelogram with length *L* = 16 mm and width *w* = 1 mm. Both ends of the YIG waveguide were covered with 30 nm thick gold to absorb SW reflections. The covered length *L*_*abs*_ at the midpoint of the waveguide was 2 mm.

Figure 1 illustrates the setup for the two-input phase interferometer. Two independent SWs were injected into the waveguide and the interference was investigated via inductive coupling to an external rf circuit. In-phase inputs gave an amplified output and anti-phase inputs gave an attenuated output, which provided two states corresponding to binary ‘1’ and ‘0’. A partially metallized 18.4 μm thick monocrystalline YIG film was used as a SW waveguide (see ‘Methods’ for sample details). This YIG waveguide was magnetized out of plane by an electromagnet, and fixed on top of three 50 μm width microstrip lines (MSLs). SWs were excited from ports 1 and 2, and the resulting SW was detected at port 3. The distances between ports 1, 2, and 3 were 5 mm. The MSLs were driven at a frequency of 4.0 GHz to excite a FV SW. The initial phase offset for port 1 was controlled by a mechanical phase shifter. The amplitude of the injected signal was 200 mV, controlled by attenuators. The temperature of the waveguide fixture was 40 °C ± 0.2 °C.

### Dispersion curve for non-magnetic metal (NM)/ferromagnetic material (FM) structure

**Figure 2:**
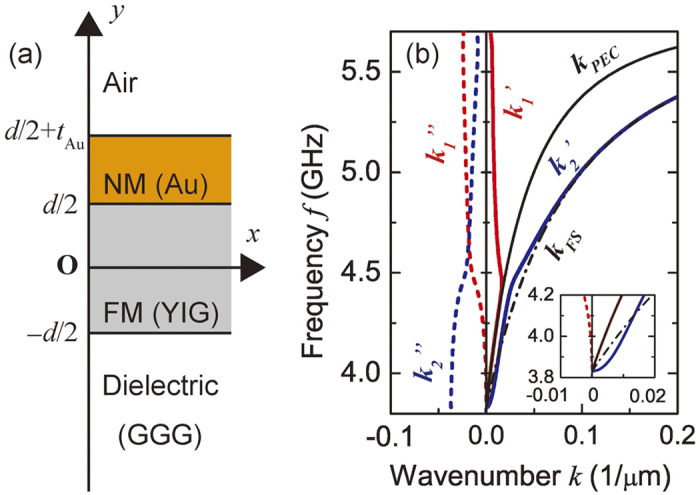
Dispersion curve of Au/YIG system. (**a**) Two dimensional infinite slab model used in the calculation. From the top, there is air above *y* = *d*/2 + *t*_Au_, nonmagnetic metal (NM, Au) between *y* = *d*/2 + *t*_Au_ and *d*/2, ferromagnet (FM, YIG) between *y* = *d*/2 and −*d*/2, and dielectric material (gadolinium gallium garnet, GGG) below *y* = −*d*/2. (**b**) Calculated dispersion curves for the Au/YIG system. Dashed black line shows the mode *k*_*FS*_ representing the dispersion curve of a free slab without an Au layer. *k*_*PEC*_ shown by the bold black line is that of a slab metallized by a perfect electrical conductor. Other lines are dispersion curves calculated for a slab metallized by 30 nm thick Au. Bold lines show the real part of the wavevectors 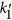 and 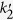. Bold dotted lines show the imaginary part of the wavevectors 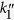 and 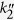.

To estimate the optimum range of frequencies and applied bias fields at which the Au layer operates as an absorber, the dispersion curve was calculated using the two dimensional infinite slab model shown in Fig. 2a. With YIG as a FM and Au as a NM, we obtained the dispersion curve using the model for effective boundary conditions (see ‘Methods’ section for theoretical details):


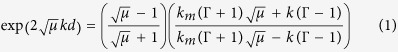



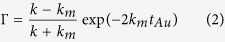



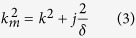


Here, *k*(*ω*) is the wavenumber of the FV SW in the Au/YIG structure, *μ*(*ω*) is the permeability of the YIG layer, *δ*(*ω*) is the skin depth of the Au layer at the frequency *ω*, and *d* = 18.4 μm and *t*_Au_ = 30 nm are the thicknesses of the YIG layer and the Au layer, respectively. Figure 2b shows the dispersion curves *k*(*ω*) calculated by equation (1) with the following set of parameters: the external magnetic field *H*_0_ = 3050 Oe, the demagnetization field *H*_*d*_ = 1682 Oe, the saturation magnetization of the YIG layer 4*πM*_*S*_ = 1760 G, Gilbert damping constant *α* = 2.35 × 10^−4^ for the YIG layer, the conductivity of Au σ_Au_ = 4.1 × 10^7 ^Sm^−1^. The solution exhibited hybridization of SW modes labeled as 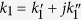 and 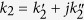. In Fig. 2b, at the lower frequencies, mode *k*_1_ resembles *k*_*PEC*_, the case of a perfect electric conductor, (i.e. *σ*_Au_ → ∞). Mode *k*_2_ resembles *k*_*FS*_, the case of a free slab (i.e. *σ*_Au_ → 0). These results therefore show that the rf field penetrating the thin Au layer allows the existence of *k*_2_ which behaves like *k*_*FS*_. Based on these results, the working frequency was fixed at 4.0 GHz at which both *k*_1_≈*k*_*PEC*_ and *k*_2_ ≈ *k*_*FS*_ were present.

### Design of SW absorber

**Figure 3:**
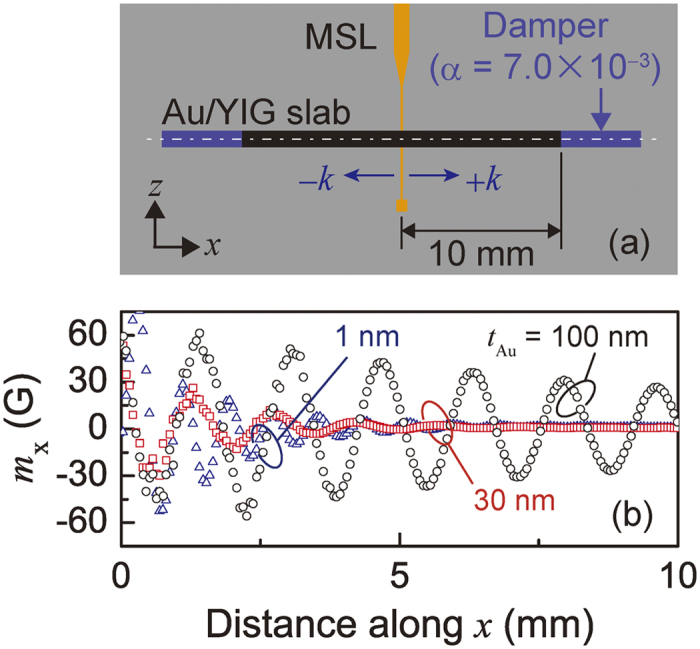
Simulation of magnetization in a pseudo-infinite Au/YIG slab. (**a**) Simulation model for FEM calculation. An infinite Au/YIG slab as shown in Fig. 2a was assumed along the *x*-direction. Edge reflection was totally cancelled by absorption boundaries colored in blue, in which a higher Gilbert damping of *α* = 7.0 × 10^−3^ was set. (**b**) Magnetization precession amplitude along the +*k* direction for different Au thicknesses. The spatial distribution of magnetization *m*_*x*_ was extracted within 10 mm of the MSL edge.

To design the length and the thickness of the Au layer of the SW absorber, the behavior of the SW was calculated by a finite element method (FEM) using the model shown in Fig. 3a. Both edges of the YIG were treated as having a non-reflective boundary condition. The YIG thickness was 18.4 μm and the Au thickness was *t*_Au_ = 1, 30 or 100 nm. A SW was excited by the MSL, and its spatial decay in the continuous slab of Au/YIG was calculated (see ‘Methods’ section). Figure 3b gives the amplitude of the SW (i.e., the magnetization *m*_*x*_ along the + *k* direction) as a function of the distance from the edge of the MSL. When *t*_Au_ = 1 nm and 30 nm, the SW decayed more rapidly and the period of oscillation increased monotonically as *t*_Au_ increased. (The mechanism for this is discussed in the next section.) Simulated wave profiles can be expressed by 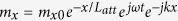 where *m*_*x*0_ is the amplitude of magnetization beneath the MSL, *L*_*att*_ is the attenuation length and *ω* is the precession frequency. For *t*_Au_ = 1, 30, and 100 nm, *L*_*att*_ was evaluated as 1.5 mm, 1.7 mm, and 3.4 mm, respectively. To suppress SW reflection, the length of the SW absorber *L*_*abs*_ should be longer than *L*_*att*_, which leads to a smaller device when the Au is thinner.

### Mechanism of SW absorption using Au film

**Figure 4:**
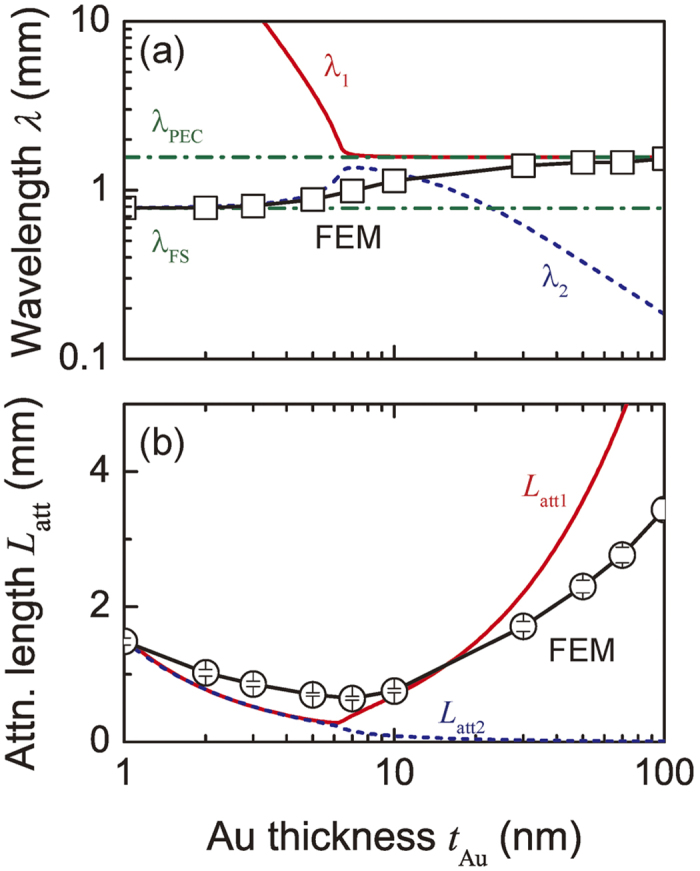
Calculated transmission properties of Au/YIG waveguide with different Au thicknesses. (**a**) Au thickness dependence of wavelength. Red bold line *λ*_1_ and blue dot line *λ*_2_ correspond to the wavelengths of modes *k*_1_ and *k*_2_ denoted in Fig. 2b. Open squares show the wavelengths obtained by the FEM calculation in Fig. 3. Green broken lines *λ*_*FS*_, *λ*_*PEC*_ show that of the free slab and the slab metallized by a perfect electric conductor. (**b**) Au thickness dependence of attenuation length. Red bold line *L*_*att* 1_ and blue dot line *L*_*att* 2_ correspond to modes *k*_1_ and *k*_2_. Open circles are the results of the FEM calculation with error bars from the exponential curve fitting of the magnetization decay.

Figure 4 shows the wavelength and the attenuation length of the FV SW vs. Au thickness for SWs propagating in the Au/YIG structures. The theoretical values of the wavelength and the attenuation length calculated from equation (1) are overlaid on the results of FEM calculation using the following relationships^32^: *λ*_1_ = 2*π*/Re(*k*_1_), *λ*_2_ = 2*π*/Re(*k*_2_), *L*_*att*1_ = 1/Im(*k*_1_), *L*_*att*2_ = 1/Im(*k*_2_). The results of the FEM calculations involved contributions from both modes *k*_1_ and *k*_2_ predicted by equation (1). Hence it is important to understand the behavior of each mode to design the SW absorber. The results of FEM calculations matched *k*_1_ when *t*_Au_ > 30 nm and *k*_2_ when *t*_Au_ < 5 nm, and exhibited intermediate values between these thicknesses, where *k*_1_ and *k*_2_ mixed to form a hybrid mode. This explains why the spatial period of the magnetization oscillation increased with the Au thickness in Fig. 3b as the propagation mode gradually transitioned from *k*_2_ to *k*_1_ through the hybrid state. The wavelength of SWs is usually extended in metallized waveguides^33^. In Fig. 4b, the shortest attenuation length occurred around *t*_Au_ ~ 6 nm where *k*_1_ and *k*_2_ were strongly hybridized, i.e. the hybridization attenuates the SW. Outside the hybrid region, i.e. *t*_Au_ < 5 nm or *t*_Au_ > 30 nm, the attenuation length increased. The Au thickness should be chosen to excite strong hybridization, leading to a choice of Au thickness below about 30 nm to get *L*_*att*_ below ~2 mm.

### Suppression of reflected SWs at the edge

**Figure 5:**
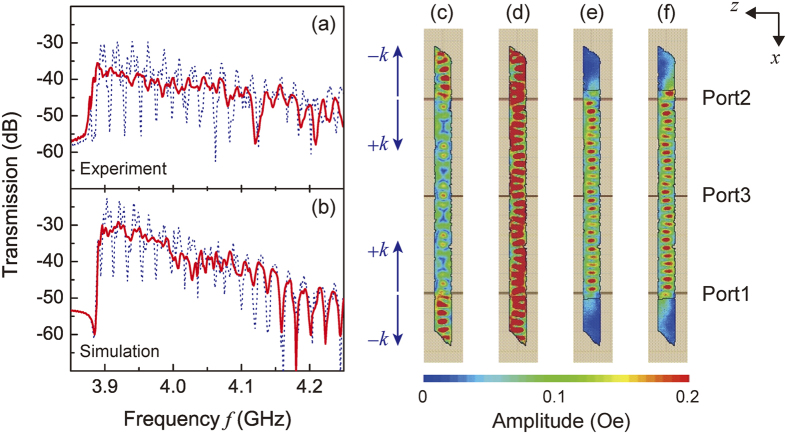
SW propagation in a two port interferometer. (**a**) Transmission properties from port 2 to port 3 in experiment under a bias magnetic field of *H*_*0*_ = 3050 Oe. (**b**) Simulated under an effective field of *H*_0_ − *H*_*d*_ = 1368 Oe. Blue dashed lines and red bold lines show transmission behavior without and with the Au absorber, respectively. (**c**) Distribution of the SW amplitude without the Au absorber at a frequency *f* = 3.998 GHz corresponding to a ripple bottom, and (**d**) at *f* = 4.001 GHz corresponding to a ripple top; (**e**) SW amplitude at 3.998 GHz and (**f**) at 4.001 GHz for the Au/YIG structure. The color represents SW amplitude. Wavenumbers + *k* and −*k* are defined as inward and outward flow at port 3.

A SW waveguide was fabricated comprising a 30 nm thick, 2 mm long Au layer deposited on each end of an 18.4 μm thick, 16 mm long YIG crystal with tapered edges, and transmission spectra between ports 1 and 3 were measured (Fig. 5a). Clearly the transmission ripples, which are due to the reflected SWs at the edge of the waveguide, were suppressed by the SW absorber compared with that of a bare YIG waveguide, in reasonable agreement with the calculated results shown in Fig. 5b. Transmission ripples observed at frequencies higher than 4.1 GHz were due to modes in the width direction, and the differences between the experimental results and simulations in this frequency range may be attributed to the inhomogeneity of the demagnetization field. The effects of the reflected SWs can be visually understood by examining the SW distribution calculated using FEM for the shape of the particular waveguide used in the experiment. Figure 5c–f show the calculated spatial amplitude distribution when the same SW was injected from Port 1 and 2, without (Fig. 5c,d) and with (Fig. 5e,f) the Au. The SW amplitude corresponds to a perturbing magnetic field |*h*_*x*_| = |−∇_*x*_*φ*|∝|*φ|*, derived using the magnetostatic approximation (the scalar potential of the SW was expressed by 
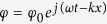
, where *t* is time). In the case of a waveguide without Au, the strong reflected SWs produced a qualitatively different amplitude distribution between frequency *f* = 3.998 GHz (bottom of ripple, Fig. 5c) and *f* = 4.001 GHz (top of ripple, Fig. 5d). In contrast, in the presence of the Au absorber, the difference between the amplitude distributions was small (Fig. 5e,f), due to the suppression of reflected SWs.

### FV SW Interference

**Figure 6:**
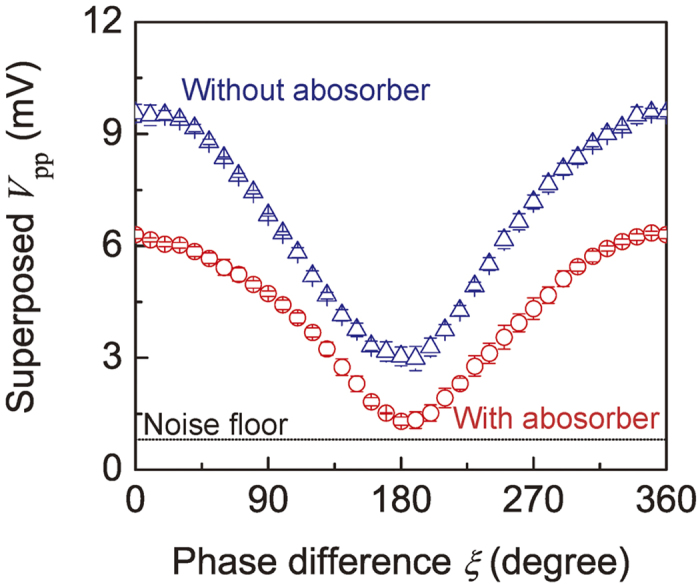
Phase interference properties of FV SW interferometer. Amplitude of the superposed output wave *V*_*pp*_ detected at port 3 versus the phase difference *ξ* between two injected waves, for YIG without Au (blue triangles) and with an Au coating (red circles). Error bars correspond to the standard deviations of five repeated measurements.

Figure 6 shows the experimental demonstration of a SW interferometer using FV modes at an operating frequency of 4.0 GHz. A fixed bias magnetic field of *H*_0_ = 3050 Oe was applied perpendicular to the film and the SW wavelength was estimated to be about 780 μm from the dispersion curve^2^. The input voltage was 200 mV for ports 1 and 2 and the output was measured at port 3.

For the device without Au, when the phase difference *ξ* between the injected waves was zero, constructive interference of the SWs led to a high output amplitude of *V*_pp_ = 9.56 ± 0.24 mV (the error represents the standard deviation of five measurements). When *ξ* = *π*, the interference was destructive and the amplitude was *V*_pp_ = 3.04 ± 0.25 mV. Consequently the isolation ratio, defined as the ratio of these amplitudes, was 9.95 dB.

**Figure 7:**
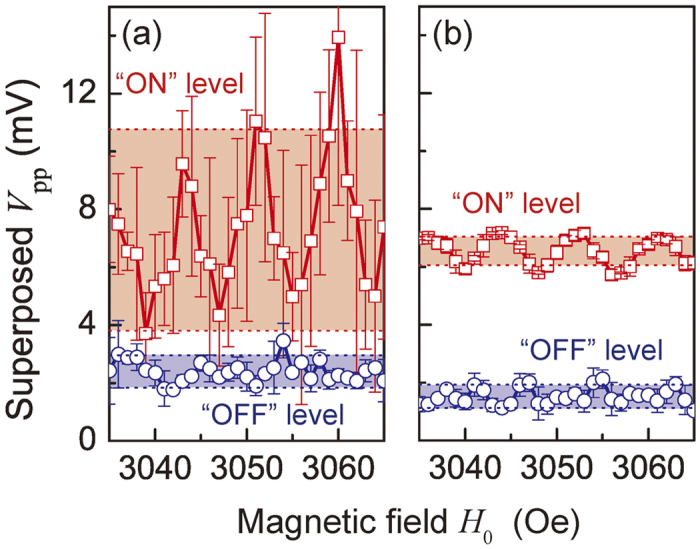
Robustness of operation against magnetic field disturbance. Red squares show the superposed output amplitudes when the injected waves are in-phase (“ON”), and blue open circles show the outputs for anti-phase injected waves (“OFF”), for the device (**a**) without and (**b**) with an Au absorber. The width of the colored band represents the standard deviation. Error bars correspond to the standard deviations of five repeated measurements at one field.

In contrast, an Au/YIG waveguide showed a higher isolation value of 13.7 dB. The output amplitude was smaller than that of the bare YIG because of the decreased amount of reflected SWs at the edges of waveguides, but this increased the robustness of the output with respect to variations in the external magnetic field. Figure 7 shows the magnetic field dependence of the output amplitude of interfering SWs in Au/YIG and YIG devices. In a practical situation, the drift of the magnetic field is likely to be less than 10 Oe, comparable with the case of using a samarium cobalt magnet to magnetize a YIG waveguide around room temperature (25–35 °C)^34^. In this measurement, the bias magnetic field was swept from 3035 Oe to 3065 Oe in 1 ± 0.1 Oe steps, while the phase difference *ξ* was fixed at *π* or 0. The averaged amplitude at *ξ* = *π* was denoted as “OFF” and that at *ξ* = 0 was “ON”. In both Fig. 7a,b, the drift in the OFF level was small because the interfering SWs were cancelled regardless of the bias magnetic field and existence of absorbers, whereas notable periodic oscillations in the ON level were caused by the transmission ripples exhibited in Fig. 5a. The standard deviation of the output signal within the measured magnetic field range from 3035 Oe to 3065 Oe was denoted as *σ*_*ON*_ and *σ*_*OFF*_. Values of *σ*_*ON*_ = 3.48 mV and *σ*_*OFF*_ = 0.56 mV were found for the waveguide without Au coating, compared to *σ*_*ON*_ = 0.50 mV and *σ*_*OFF*_ = 0.40 mV in the presence of the Au, shown in Fig. 7b. Colored areas indicate *σ*_*ON*_ and *σ*_*OFF*_. Without Au, the discrimination between ON and OFF levels was only 0.85 mV, whereas the Au/YIG device showed ON and OFF levels well separated by 4.14 mV, which exceeded the standard deviation of the output signal. These results indicate that a SW interferometer with an edge absorber comprising an Au/YIG structure ensured a robust and reliable operation over a magnetic field range of tens of Oe, a significant advance towards the practical implementation of FV SW logic devices. For further miniaturization and integration of such a device, the contribution of electromagnetic noise may be non-negligible and further improvement of the amplitude ratio between ON and OFF states will be significant. This will be achieved by replacing MSLs with nanoscale coplanar waveguides with a meander structure, in which direct waves between antennas are diminished and FV SWs are efficiently excited.

## Conclusion

We experimentally demonstrated a FV SW phase interferometer based on a low damping YIG waveguide. An Au SW absorber suppressed SW reflection from the ends of the device and increased the signal to noise ratio such that the ON and OFF output signals are well discriminated even for significant variations in the bias magnetic field. The SW propagation and interference in the waveguide was analyzed with a finite slab model and three dimensional FEM calculations, which enabled the device geometry to be designed. Excitation of a hybrid SW mode at intermediate Au thicknesses was essential for stable operation of SW interferometers. Interference of two input SWs produced a binary output with a large amplitude ratio of 13.7 dB between ON and OFF states. The SW interference was comparable with other SW interferometers using BV or DE SW modes^21^,^22^,^23^, but unlike the other SW modes, the use of FV SWs has great benefits for the circuit integration because of the spatial isotropy of wave propagation. This study represents the first important step for building ultralow-power integrated circuits while taking advantage of both the low damping of YIG waveguides and the isotropic propagation of FV SWs.

## Methods

### Sample preparation and characterization

Our SW waveguide comprised an 18.4 μm-thick YIG, which was grown on a gadolinium gallium garnet (GGG) substrate by liquid phase epitaxy. The surface of the waveguide was polished to yield a roughness *R*_*a*_ = 0.4 nm so that resonant coupling with a perpendicular standing SW was reduced. The YIG wafer was cut into *L* = 16 mm by *w* = 1 mm with 45° tapered edges formed by dicing. Both ends of the YIG waveguide were covered by a 30 nm thick continuous Au film, with *R*_*a*_ = 0.4 nm, deposited by rf magnetron sputtering (Shimazu, HSR-551). The *R*_*a*_ values were measured by stylus profilometer (Ryokosha Corp., ET4000).

The Gilbert damping factor *α* of the film was evaluated by a standard vector network analyzer (VNA)–ferromagnetic resonance (FMR) setup^35^. The YIG specimen was cut into a 1.3 mm by 1.3 mm square and placed on a 50 Ω matched microstrip-through line with a length of 40 mm. A bias magnetic field *H*_0_ was applied perpendicular to the film plane, and scattering parameters were measured by the VNA (Anritsu 37347C). Then the permeability around the resonance peak was calculated and its line width Δ*f* was obtained. In general, the measured line width Δ*f* is composed of two contributions: intrinsic damping *α* and extrinsic damping Δ*H*_0_ 36,


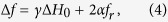


where *γ* = 2.8 MHz/Oe is the gyromagnetic ratio and *f*_*r*_ is the FMR frequency. Extrinsic damping is considered as an extra contribution from, for example, inhomogeneity of the rf excitation field from the microstrip-through line. Such extrinsic contributions were eliminated by extracting the slope of Δ*f* vs. *f*_*r*_. The FMR frequency was swept from 3 GHz to 4 GHz by changing the bias magnetic field *H*_0_ from 2760 Oe to 3140 Oe in 20 Oe steps. The intrinsic damping of the YIG film was estimated as *α* = 2.35 × 10^-4^. This value was used in all calculations in this study. In addition, spin pumping at the Au/YIG boundary provided another contribution to the damping. The extra damping *α*_*sp*_ from spin pumping was estimated from the formula^37^


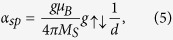


where *g* = 2 is the Landé g-factor, *μ*_*B*_ = 9.274 × 10^−21 ^erg/G is the Bohr magneton, 4*πM*_*S*_ = 1760 G is the saturation magnetization of YIG, *g*_↑↓_ = 1.2 × 10^14 ^cm^−2^ is the spin mixing conductance at the Au/YIG boundary^38^ and *d* = 18.4 μm is the thickness of YIG. The estimated *α*_*sp*_ = 6.87 × 10^−7^ was much smaller than the intrinsic damping because the YIG film was thick, and the spin pumping contribution was therefore neglected.

### Calculation of dispersion curve

In the calculation of dispersion curve shown in Fig. 2, we used a state vector (*h*_*x*_, *b*_*y*_) where *h*_*x*_ is the *x*-component of the magnetic field perturbation and *b*_*y*_ is the *y*-component of the magnetic flux perturbation^39^. Since the slab is homogeneous along the *z*-direction, ∂/∂*z* = 0 for all layers. At each boundary, the continuity of the state vector was ensured. The magnetostatic approximation ∇ × *h* = 0 was applied except for the NM layer^1^. In the other layers, the SW can be expressed in the form of a scalar potential 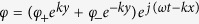 with eigenvalues *φ*_+_ and *φ*_−_. The values of *h*_*x*_ and *b*_*y*_ were obtained from *h*_*x*_ = −∇_*x*_*φ* and *b*_*y*_ = [−**μ**∇*φ*]_*y*_ where **μ** is the permeability tensor when the mean magnetization is along the *y*-direction. In the NM layer, a quasi-steady approximation was used to consider the effect of the finite conductivity *σ*_*NM*_. The state vector in this layer was obtained by solving ∇ × *h* = *σ*_*NM*_*E* for *h*_*x*_ and *h*_*y*_, where *E* is the electric field. The magnetic flux *b*_*y*_ (G) is numerically equal to *h*_*y*_ (Oe) because the layer is defined as non-magnetic. Solving the set of equations for the eigenvector (*φ*_+_, *φ*_−_) gave a characteristic equation, a function of wavenumber *k* and frequency *ω*, and the non-trivial solution for the eigenvector determines the dispersion curve. The demagnetization field *H*_*d*_ = 1682 Oe was obtained by the fitting of the measured phase spectrum of the propagating FV SW *θ* = −*kL*_*p*_ as a function of *ω* and *H*_0_ under the bias magnetic field *H*_0_ = 3050 Oe with a propagation length *L*_*p*_ = 10 mm. The wavenumber *k* in the YIG waveguide (free slab) can be calculated by substituting *t*_Au_ = 0 or *δ* → ∞ (meaning *σ*_Au_ → 0) into equation (1). This nonlinear dispersion equation was numerically solved by the Levenberg-Marquardt method in MATLAB.

### Estimation of the attenuation length

ANSYS HFSS version 15 was used for the simulation shown in Fig. 3 and parts of Fig. 4. The simulation model was composed of a 1 mm-wide and 30 mm-long Au/YIG multilayer with the YIG in contact with the MSL. To realize an infinite slab in the FEM calculation, edge reflection was excluded. The magnetic damping of YIG at both waveguide edges was set to a large *α* = 7.0 × 10^−3^ to eliminate any reflected waves (blue colored region in Fig. 3a). This is a convenient way to investigate the pure SW transmission properties in Au/YIG multilayers. The spatial distribution of the *x*-axis component of the magnetization *m*_*x*_ was then calculated for various Au layer thicknesses. The distribution of the amplitude was extracted at the central width of the waveguide. The spatial decay of the envelope of magnetization *m*_*x*_ along + *k* was fitted to exp (−*x*/*L*_*att*_) which yielded the attenuation length *L*_*att*_. The wavenumber *k* was evaluated by Fourier transformation of the spatial oscillation of *m*_*x*_.

## Additional Information

**How to cite this article**: Kanazawa, N. *et al.* Demonstration of a robust magnonic spin wave interferometer. *Sci. Rep.* **6**, 30268; doi: 10.1038/srep30268 (2016).
